# FACTORS ASSOCIATED WITH HAND GRIP STRENGTH AMONG OLDER ADULTS—FINDINGS FROM THE LASI STUDY INDIA

**DOI:** 10.1093/geroni/igad104.2929

**Published:** 2023-12-21

**Authors:** Snehal Kulkarni

**Affiliations:** Dr. Vishwanath Karad’s MIT-World Peace University, Pune, Maharashtra, India

## Abstract

**Objective:**

To identify the factors associated with handgrip strength among older adults in India.

**Methods:**

Data for the study were drawn from the baseline survey of Longitudinal Ageing Study in India (LASI, 2017–18). This study was conducted on respondents aged 60 years and above with a total sample of 27,347 older adults (men-13,241 and women-14,118). The study assessed handgrip strength using a handheld Smedley’s Hand Dynamometer. Descriptive statistics and independent t-test was used to measure the mean difference in the handgrip strengths according to presence of risk factors.

**Results:**

The mean handgrip strength was 29.76 + 11.23 kgs for both the genders. There was statistically significant difference in the mean handgrip strength of men (36.26 + 10.83 kg) and women (23.70 + 7.65 kg respectively). Significantly lower handgrip strength was observed among participants in rural area (29.58 + 11.20 kg), difficulty in activities of daily living (28.24 + 10.97 kg), low level physical activity, balance impairment (21.09 + 8.83 kg) slowness in walking (25.14 + 9.87 kg), history of falls (28.24 + 10.97 kg), and presence of bone and joint disease (27.44 + 10.60 kg) as compared to their counterparts.

**Conclusion:**

The study contributed to a better understanding of factors associated with HGS, and thus, the HGS is recommended as a section in the health evaluation among high-risk older adults as the strategy of disease control and prevention.

